# Identification of novel vibration- and deflection-sensitive neuronal subgroups in Johnston's organ of the fruit fly

**DOI:** 10.3389/fphys.2014.00179

**Published:** 2014-05-09

**Authors:** Eriko Matsuo, Daichi Yamada, Yuki Ishikawa, Tomonori Asai, Hiroshi Ishimoto, Azusa Kamikouchi

**Affiliations:** ^1^Division of Biological Science, Graduate School of Science, Nagoya UniversityNagoya, Japan; ^2^Precursory Research for Embryonic Science and Technology, Japan Science and Technology AgencyTokyo, Japan

**Keywords:** Johnston's organ, *Drosophila*, brain, calcium imaging, neural circuit, mechanosensory, insect

## Abstract

The fruit fly *Drosophila melanogaster* responds behaviorally to sound, gravity, and wind. Johnston's organ (JO) at the antennal base serves as a sensory organ in the fruit fly to detect these mechanosensory stimuli. Among the five anatomically defined subgroups of sensory neurons in JO, subgroups A and B detect sound vibrations and subgroups C and E respond to static deflections, such as gravity and wind. The functions of subgroup-D JO neurons, however, remain unknown. In this study, we used molecular-genetic methods to explore the physiologic properties of subgroup-D JO neurons. Both vibrations and static deflection of the antennal receiver activated subgroup-D JO neurons. This finding clearly revealed that zone D in the antennal mechanosensory and motor center (AMMC), the projection target of subgroup-D JO neurons, is a primary center for antennal vibrations and deflection in the fly brain. We anatomically identified two types of interneurons downstream of subgroup-D JO neurons, AMMC local neurons (AMMC LNs), and AMMC D1 neurons. AMMC LNs are local neurons whose projections are confined within the AMMC, connecting zones B and D. On the other hand, AMMC D1 neurons have both local dendritic arborizations within the AMMC and descending projections to the thoracic ganglia, suggesting that AMMC D1 neurons are likely to relay information of the antennal movement detected by subgroup-D JO neurons from the AMMC directly to the thorax. Together, these findings provide a neural basis for how JO and its brain targets encode information of complex movements of the fruit fly antenna.

## Introduction

Fruit flies and other animals rely on various sensory modalities, such as olfactory, gustatory, tactile, auditory, and visual systems, to implement appropriate adaptive behaviors (Ebbs and Amrein, 2007; Kamikouchi et al., 2009; Yorozu et al., 2009; Grosjean et al., 2011; Tuthill et al., 2013). Johnston's organ (JO), the antennal ear of the fruit fly (Göpfert and Robert, 2003; Tauber and Eberl, 2003; Kamikouchi et al., 2006; Albert et al., 2007; Nadrowski et al., 2011), serves as a sensor for various types of mechanosensory stimuli, i.e., sound, gravity, and wind (Kamikouchi et al., 2009; Yorozu et al., 2009). These types of mechanosensory stimuli induce particular behavioral responses in the fruit fly *Drosophila melanogaster*, e.g., exposure to male courtship songs leads to behavioral changes in males and females; when agitated, fruit flies show negative-gravitaxis behavior; and when faced with gentle air currents, fruit flies stop walking (Kamikouchi et al., 2009; Yorozu et al., 2009).

**Figure 1:**
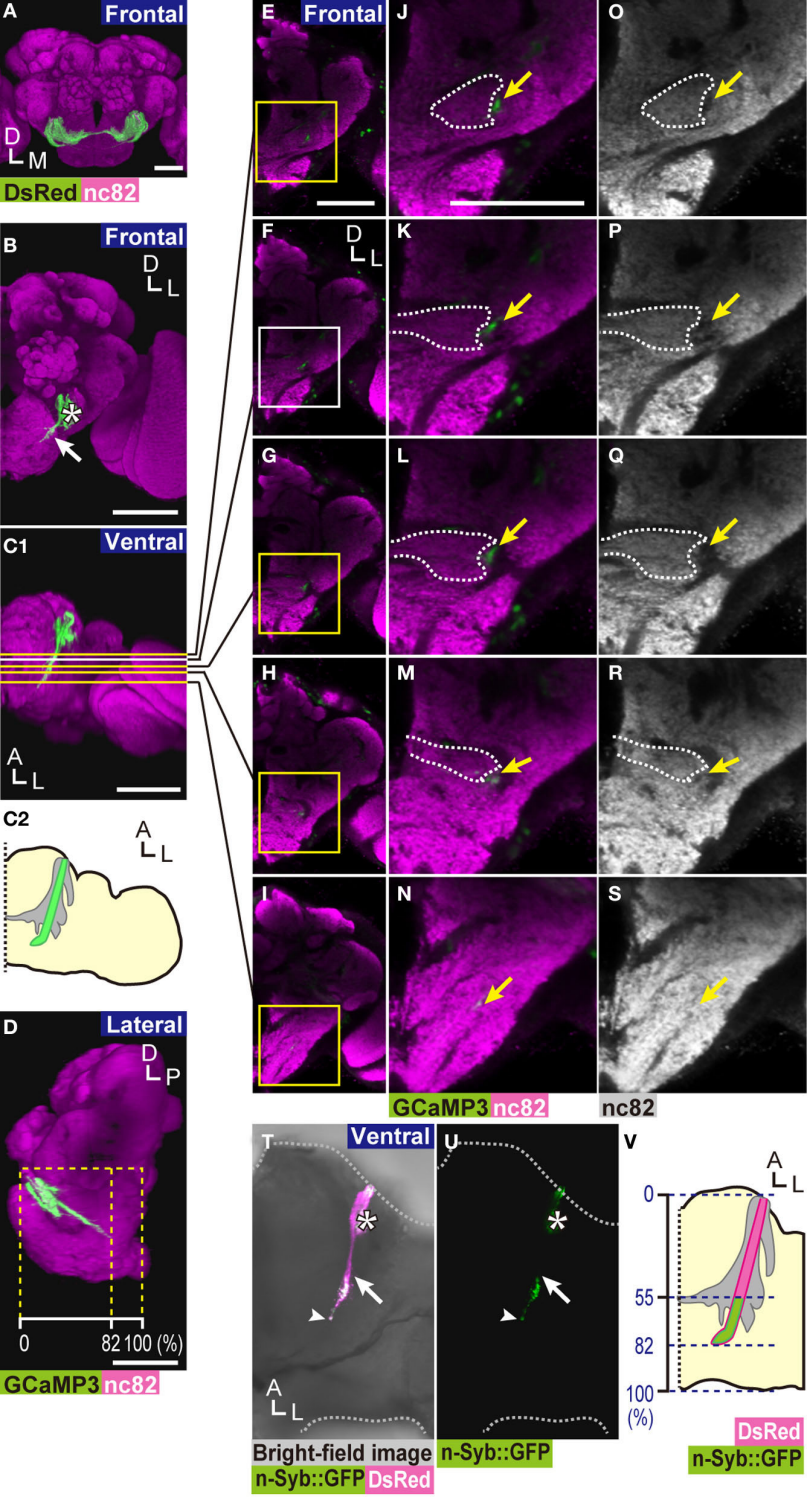
**Location of zone D in the brain. (A)** 3D-reconstructed frontal image of a fly brain. DsRed marker proteins were expressed in essentially all JO neurons using the *F-GAL4* driver (Kim et al., 2003). The AMMC is shown in green. The neuropils of a brain hemisphere were labeled with nc82 antibody (magenta). **(B)** 3D-reconstructed frontal image of the axon bundle of subgroup-D JO neurons (solid arrow) labeled by a combination of JO29 *GAL4* driver and *UAS-GCaMP3* marker strain (green). Note that JO29 *GAL4* driver also labels a few JO neurons of subgroups B and -E (asterisk). **(C)** Ventral view of **(B)**. **(C1)** Yellow and white lines indicate the position of sections shown in panels **(E–I)**. Axons of subgroup-D JO neurons start bending toward the midline of the brain at around panel **(F)** (white line). **(C2)** The schematic drawing of **(C1)**. Green zone indicates the axons of subgroup-D neurons. Gray area indicates the AMMC. **(D)** Lateral view of **(B)**. Yellow dotted lines indicate the length ratio along the anterior-posterior axis of the brain. **(E–I)** Frontal views of the brain at various depths from the anterior side of the brain (yellow and white lines in **C1**). **(J–N)** Magnified view of zone D and its surrounding area (yellow and white boxes in **E–I**). **(O–S)** A landmark structure of zone D. The same positions as in panels **(J–N)**. Yellow arrows indicate the tract in which subgroup-D axons are located **(J–S)**. White dotted lines indicate the boundary of zone E, identified by the staining pattern of nc82 antibody **(J–M,O–R)**. The axon bundle of subgroup-D JO neurons (green in panels **B–N**), nc82 (magenta in panels **A–N**, white in panels **O–S**). **(T,U)** Presynaptic sites of subgroup-D JO neurons. n-Syb::GFP signals (green) were localized mainly at the posterior side of zone D. White arrows indicate the most anterior position of strong n-Syb::GFP signals on the axons of subgroup-D JO neurons. White arrowheads indicate the terminal of zone D. Asterisks show signals derived from subgroup-B JO neurons, which are also labeled by the JO29 *GAL4* driver. **(V)** Distribution of presynaptic sites of subgroup-D JO neurons. Blue dotted lines indicate the length ratio along the anterior-posterior axis of the brain. Scale bar = 50 μm. A, anterior; P, posterior; D, dorsal; M, medial; L, lateral.

The JO sensory neurons are anatomically divided into five subgroups, subgroups A–E (Kamikouchi et al., 2006). The axons of each neuronal subgroup project to a specific zone (zones A–E) in the antennal mechanosensory and motor center (AMMC), which is located on the ventral side of the brain (Figure 1A). Subgroup-A and -B JO neurons are vibration-sensitive neurons and project to zones A and B in the AMMC, respectively. Subgroup-C and -E JO neurons, which innervate zones C and E in the AMMC, respectively, selectively respond to static deflections of the antennal receiver. The expression of neural toxins in a spatiotemporally controlled manner can selectively and transiently block the function of the JO neuronal subgroups (Kamikouchi et al., 2009). Findings from such experiments indicate that vibration-sensitive neurons are required for hearing, and deflection-sensitive neurons are required for gravity and wind sensing (Kamikouchi et al., 2009; Yorozu et al., 2009).

The number of subgroup-D JO neurons is estimated to be approximately 40 (Kamikouchi et al., 2006). These neurons project to the posterior side of the brain so called zone D in the AMMC, with some of them also projecting a collateral to zones A and/or B. Subgroup-D JO neurons has a characteristic anatomy when compared with other JO subgroups; zone D protrudes to the most posterior region of AMMC with little arborization, unlike other zones in the AMMC. Moreover, cell bodies of subgroup-D JO neurons distribute as a pair of clusters in JO whereas cell bodies of other subgroups, subgroups A, B, and C and E, distribute in a ring-like shape with partially overlapping manner (Kamikouchi et al., 2006; Matsuo and Kamikouchi, 2013). Such characteristic anatomy of subgroup-D JO neurons would possibly be related to their unique function in JO neurons subgroups. The response properties of subgroup-D JO neurons, however, remain unclear. To address this question, we performed GCaMP3 calcium imaging combined with an electrostatic method to precisely control the movement of the antennal receiver. Here we report the response properties of subgroup-D JO neurons. We also observed distinct types of interneurons that connect zone D to other regions of the central nervous system.

## Materials and methods

### Experimental animals

Female fruit flies *D. melanogaster* aged between 5 and 10 days after eclosion and raised on standard *Drosophila* yeast-based media at 25°C at 40 to 60% relative humidity were used. The following transgenic *GAL4*-driver strains were used for the GAL4/UAS (Brand and Perrimon, 1993) and FLP-out (Basler and Struhl, 1994) techniques: *F-GAL4* (Kim et al., 2003) and JO29 (Kamikouchi et al., 2006) for calcium imaging; and NP2228, NP5056, and NP7365 (*Drosophila* Genetic Resource Center, Kyoto, Japan) for the FLP-out analysis to generate samples for visualizing single neurons. The following *UAS-reporter* strains were used: *UAS-neuronal synaptobrevin::GFP* (*n-syb::GFP*; Ito et al., 1998; Estes et al., 2000), *UAS-DsRed S197Y* (Verkhusha et al., 2001), and *UAS-GCaMP3* (Bloomington Stock Center, Bloomington, IN). Flies carrying the transgenes *hs*-*flp* and *UAS* >*CD*2, *y*^+^ >CD8::GFP (Wong et al., 2002) were used for the FLP-out analysis. To visualize single neurons, flies from 1 to 2 days after eclosion were placed in a plastic tube at 37°C for 5 min to induce flippase expression. For the Ca^2+^ imaging and neuroanatomic analyses, the flies were made homozygous for both *GAL4* and *UAS-GCaMP3*. For mechanical measurements of the antennal receiver, *tilB*^1^ mutants obtained by selecting hemizygous males from the balanced stock *yw tilB*^1^/*FM4* (Kavlie et al., 2010) were used.

### Immunohistochemistry

Immunostaining of the fly brain was performed as described previously with minor modifications (Kamikouchi et al., 2006). Briefly, the brains were dissected in phosphate-buffered saline (PBS, pH 7.4 at 25°C), fixed with 4% paraformaldehyde in PBS for 90 min on ice, incubated overnight at 4°C in PBS containing 0.5% Triton X-100 (PBT), and stained by antibodies. The following antibodies were used: anti-GFP (rabbit polyclonal IgG, 1:1000, Invitrogen, Carlsbad, CA) to enhance the GFP signal of GCaMP3 and CD8::GFP, anti-DsRed (rabbit polyclonal, 1:1000, Clontech Laboratories, Inc., Mountain View, CA) to enhance the DsRed S197Y signal, and anti-Bruchpilot, nc82 (mouse monoclonal IgG, 1:20, Developmental Studies Hybridoma Bank, Iowa City, IA) to visualize brain areas rich with synapses. Alexa Fluor 555-conjugated anti-rabbit IgG (1:300, Invitrogen), and Alexa Fluor 647-conjugated anti-mouse IgG (1:300, Invitrogen) were used as secondary antibodies. After rinsing with PBT and PBS, samples were incubated in 80% glycerol in deionized water overnight and mounted on glass slides (Matsunami Glass IND., LTD, Osaka, Japan). Brains without immunolabeling (Figures 1T,U) were mounted on slides immediately after dissection.

### Confocal microscopy and image processing

Serial optical sections were obtained at 0.84-μm intervals with an FV-1000D laser-scanning confocal microscope (Olympus, Tokyo, Japan) equipped with a silicone-oil immersion 30× Plan-Apochromat objective lens (NA = 1.05). For three-dimensional (3D) image reconstruction, confocal image datasets were processed with the 3D-reconstruction software FluoRender (Wan et al., 2009; http://www.fluorender.org). For the projection analysis and FLP-out image analysis, signals of cells that were not relevant to the traced neurons were erased manually from the original images with FluoRender for clarity. The size, contrast, and brightness of the images were adjusted using Photoshop CS5 (Adobe Systems, San Jose, CA). Projection patterns for each neuron were analyzed in at least three animals to obtain consistent images across individuals for Figures 1B–V, 5. To map the positions of nSyb::GFP signals and dendritic regions of the AMMC LNs and AMMC D1 along the anterior-posterior axis of the brain, confocal image datasets were 3D reconstructed on FluoRender software. After their angles were adjusted to give the ventral view of the brain, the distance between the entrance point of the antennal nerve to the brain and the following three points were measured manually: the posterior end of the central brain, distribution area of nSyb::GFP signals, and the position where dendritic regions of the AMMC LNs and AMMC D1 were observed. Neuropils were defined according to the systematic nomenclature of the insect brain proposed by the Insect Brain Name Working Group (Ito et al., 2014).

### Calcium imaging

To enhance the GCaMP3 expression, 2- to 7-day old female flies were incubated with fly food at 29°C for 24 h. After incubation, each fly was anesthetized on ice and affixed onto an imaging plate using silicon grease (SH 44M, Toray, Tokyo, Japan) with the ventral side of the fly up. The mouthpart of the fly was then removed using fine tweezers under a stereomicroscope (M125, Leica Microsystems GmbH, Wetzlar, Germany) to open a window through which we could monitor brain fluorescence. A 35-mm lumox-film bottom dish (SARSTEDT AG & Co, Nümbrecht, Germany) with a square hole (0.6 × 1.0 mm) at the bottom was placed above this window of the fly head. A drop of *Drosophila* Ringer's solution (130 mM NaCl, 5 mM KCl, 2 mM CaCl_2_, 2 mM MgCl_2_, 36 mM sucrose, and 5 mM HEPES [pH 7.3]) (Fiala and Spall, 2003) was immediately added to prevent dehydration. After removing the small trachea and excessive fat with fine tweezers, the fly, together with the imaging plate, was fixed to the stage of a fluorescent microscope (Axio Imager.A2, Carl Zeiss, Oberkochen, Germany) equipped with a water-immersion 20× objective lens (NA = 0.5) and a spinning disc confocal head CSU-10 or CSU-W1 (Yokogawa, Tokyo, Japan). This system was equipped with a krypton/argon laser for excitation at 488 nm, a dichroic beam-splitter 405/488/561/640 Di01-T405/488/568/647-13 × 15 × 0.5 (Semrock, Rochester, NY), and a band-path filter FF01-528/38-25 (Semrock). The fluorescent image was captured at a rate of 3 Hz with an exposure time of 300 ms using an EM-CCD camera (ImagEM512, Hamamatsu Photonics, Shizuoka, Japan) in water-cooled mode. Each experiment was performed in at least seven flies. The image data were analyzed offline with ImageJ (National Institutes of Health) and Excel (Microsoft Corporation) software. Images were corrected for the animal's movement by using the ImageJ plug-in TurboReg (http://bigwww.epfl.ch/thevenaz/turboreg/). Regions of interest for each zone in the AMMC were chosen where abundant output synapses were observed (Kamikouchi et al., 2006; Figure 1). The GCaMP3 fluorescence intensities were normalized to those preceding the stimulus onset (*t* = −2 s). Pseudocolor images of Δ*F/F* intensity maps were generated using ImageJ.

### Electrostatic actuation of the antennal receiver

Antennal displacement was induced by electrostatic force generated by electrodes (Albert et al., 2007; Kamikouchi et al., 2009, 2010; Effertz et al., 2012). The electrical potential of the fly was increased to +15 V against ground via a charging electrode (a 0.03-mm diameter tungsten wire, Nilaco, Japan) inserted into the thorax. The following voltage commands were used: (1) sinusoids of various frequencies and continuous pulses carrying a 35-ms inter-pulse interval (IPI) that typically activates the courtship behavior of male flies (Yoon et al., 2013), ranging from −14 to +14 V and (2) positive and negative steps, −50 and +50 V. These stimuli were fed for 4 s to a stimulus electrode (a 0.3-mm diameter platinum wire, Nilaco, Japan) placed in front of the arista, the antennal receiver of the fruit fly. The distance between the arista and the stimulus electrode, measured on the image captured by an EM-CCD camera (ImagEM512, Hamamatsu Photonics) just before the calcium imaging, was kept about 300 μm. These electrical signals were generated with a data acquisition unit (Micro1401, Cambridge Electronic Design, Cambridge, UK) operated by Spike2 software (Cambridge Electronic Design), amplified by a custom-made amplifier, and fed into a stimulus electrode. To measure the stimulus-induced vibrations of a passive object, antennal vibrations of *tilB*^1^ mutant flies (Riabinina et al., 2011) to the electrostatic actuation was measured by using a Polytec NLV-2500 scanning laser Doppler vibrometer with a VIB-A-20 × LENS close-up lens (Polytec Japan, Yokohama, Japan); the fly was affixed on top of a holder, with their heads, mouthparts, wings, halteres, and legs being stabilized by wax as described previously (Göpfert and Robert, 2002). The electrical potential of the fly was then increased to +15 V against ground via a charging electrode (a 0.03-mm diameter tungsten wire, Nilaco, Japan) inserted into the thorax. Voltage command ranging from −14 to +14 V that vibrates at 40, 100, 200, 400, and 800-Hz frequencies were fed to a stimulus electrode (a 0.3-mm diameter platinum wire, Nilaco, Japan). Because it turned out to be difficult with our setup to focus the laser at the very tip of the arista, we measured vibrations at the midpoint of the arista, which vibrates at the same frequency as its tip and ~50% of previously reported amplitude measured at the tip of the arista (Göpfert and Robert, 2002). Signals were sampled at a rate of 10 kHz, subjected to fast Fourier transform analysis off-line, and filtered out the mains frequency (60 Hz). The amplitude of vibrations and static deflections on the setup for calcium imaging was adjusted to displace the arista by approximately 10 and 25 μm, respectively (the average of five animals), in which the amplitude of deflections was measured on images captured by using an EM-CCD camera (ImagEM512, Hamamatsu Photonics) while vibrating at 100-Hz frequency and statically deflecting the arista, respectively. We used this medium displacement of the arista to monitor the calcium response to static deflections because small displacement of the arista hardly induced the robust calcium response in JO neurons (Kamikouchi et al., 2009).

### Statistical analysis

Statistical analyses were performed by using Microsoft Excel software. Statistical analysis for calcium imaging of the AMMC zones to sinusoidal vibrations and static deflections was carried out using Friedman's test followed by Scheffe's multiple comparison with respect to each zone. Statistical analysis for calcium imaging of the AMMC zones to pulse song was performed by Mann-Whitney U test with respect to each zone. A significance level of 0.05 was used for all tests.

## Results

### Fine anatomy of the AMMC zone D in the brain

We previously reported the anatomy of zone D, the projection target of subgroup-D JO neurons, in the *D. melanogaster* brain (Kamikouchi et al., 2006), as a part of the AMMC. To identify a landmark to define the location of zone D precisely, we used the GAL4/UAS binary expression system in which expression of a reporter gene fused under the *UAS* is specifically activated in cells that express a yeast transcription factor GAL4 (Brand and Perrimon, 1993). The marker protein GCaMP3 (Tian et al., 2009) was selectively expressed in subgroup-D JO neurons using the JO29 fly strain as a *GAL4* driver (Kamikouchi et al., 2006). The labeled brain was then counterstained with anti-Bruchpilot nc82 antibody, a neuropil marker that labels the presynaptic active zone and thus defines the boundary of each neuropil in the fly brain (Wucherpfennig et al., 2003; Kittel et al., 2006; Wagh et al., 2006). The axon bundle of subgroup-D JO neurons innervates the most posterior area of the ipsilateral AMMC (Figures 1B–S). The main axons of subgroup-D JO neurons project posteriorly, spanning approximately 80% of the length of the anterior-posterior axis of the brain (dotted line in Figure 1D). In the AMMC where zone E (dotted lines in Figures 1J–M,O–R) is confined in the ipsilateral side of the brain, nc82 antibody poorly labeled the area occupied by subgroup-D JO neuronal axons (arrows in Figures 1J,O). More posteriorly, where zone E forms the commissure of JO neurons (Kamikouchi et al., 2006), sparse nc82 signals co-localized with zone D (Figures 1K–M,P–R). At the axon terminal of subgroup-D JO neurons, zone D co-localizes well with nc82 signals, showing its abundant presynaptic sites (Figures 1N,S). These findings suggest that output sites of subgroup-D JO neurons axons are distributed more abundantly at the posterior side of zone D.

To directly visualize presynaptic sites of subgroup-D JO neurons, we expressed two reporter genes simultaneously in subgroup-D JO neurons: neuronal synaptobrevin::GFP fusion protein (n-Syb::GFP), which targets the synaptic vesicles (Estes et al., 2000), and DsRed S197Y (Verkhusha et al., 2001), which diffuses freely in the cytoplasm (Figures 1T–V). The n-Syb::GFP signal was detected preferentially at the posterior side of zone D, spanning between 55 and 82% of the length of the anterior-posterior axis of the brain (the average of five samples) (Figures 1T,V). The JO29 *GAL4* driver also labeled a few neurons projecting to zones B and E, i.e., subgroup-B and -E JO neurons, respectively (asterisk in Figures 1B,T,U). Output sites of subgroup-B JO neurons distribute throughout zone B (Kamikouchi et al., 2006), thus the n-Syb::GFP signal observed at the anterior side of the brain (asterisks in Figures 1T,U) derived from subgroup-B JO neurons. Taken together, our finding clearly indicated that the output sites of subgroup-D JO neurons are distributed mainly at the posterior side of zone D in the brain.

### Activity imaging of JO neurons in the brain with electrostatic actuation of the antennal receiver

**Figure 2:**
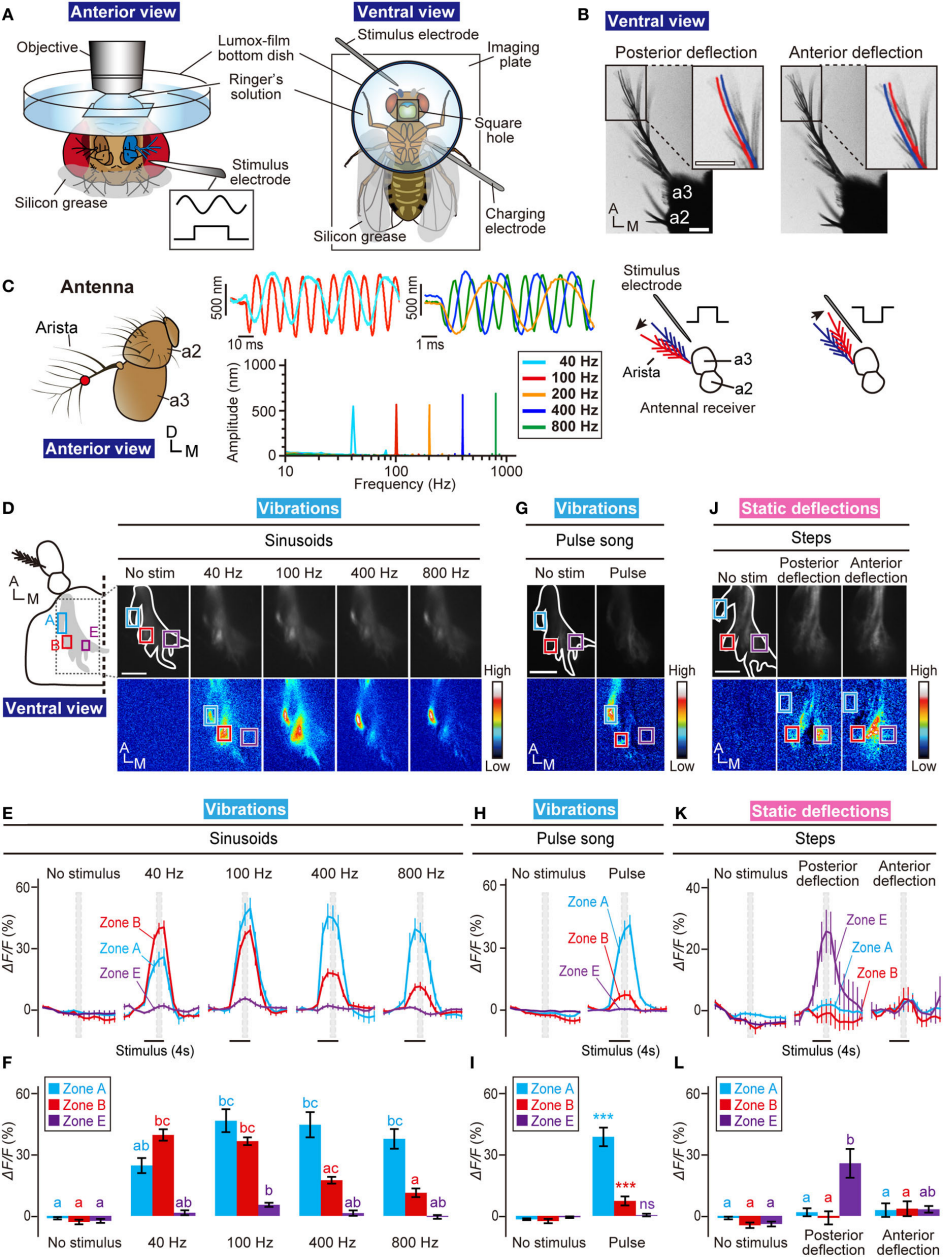
**Calcium imaging for the AMMC zones. (A)** Fly preparation to monitor calcium responses in the AMMC. A fly expressing GCaMP3 protein was fixed on a stage of a laser-scanning microscope. **(B)** Actuation of the antennal receiver. Stimulus of an electrostatic force with a positive step deflects the receiver posteriorly (Left, posterior deflection), whereas an electrostatic force with a negative step deflects the receiver anteriorly (Right, anterior deflection). Blue and red lines indicate the resting and actuated positions of the arista, respectively. a2, the second antennal segment; a3, the third antennal segment. Scale bar = 25 μm. **(C)** Vibrations of passive receivers of *tilB*^1^ mutant flies induced by an electrostatic force between 40- and 800-Hz sinusoids. Left: Frontal view of the antenna (a2, the second antennal segment; a3, the third antennal segment). Red circle shows the measurement point. Right: Time trace of the displacement at the beginning of the stimulus (top, average traces of 2–6 animals) and the mean fast Fourier transform amplitude during the stimulus (bottom, average of 6 animals) to 40, 100, 200, 400, and 800-Hz sinusoids. **(D)** Calcium responses to sinusoidal vibrations (40, 100, 400, and 800 Hz) in the AMMC. Blue, red, and magenta boxes indicate the regions of interest in zones A, B, and E, respectively, for calculating changes in fluorescent intensities shown in panels **(E,F)**. **(E)** Time-course of fluorescent changes to sinusoids in the AMMC zones A (blue), B (red), and E (magenta). Gray hatched boxes indicate the time-windows for calculating Δ*F/F* in no-stimulus (control) and during-stimulus periods shown in panel **(F)**. *N* = 12 animals. **(F)** Response properties of the AMMC zones A (blue), B (red), and E (magenta) to sinusoids. **(G)** Calcium responses to a courtship pulse song in the AMMC. **(H)** Time-course of fluorescent changes to a courtship pulse song in each zone (blue, red, and magenta boxes in panel **G**). *N* = 11 animals. **(I)** Response properties of each zone to a courtship pulse song. **(J)** Calcium responses to static deflections (anterior and posterior deflections) in the AMMC. **(K)** Time-course of fluorescent changes to static deflections in each zone (blue, red, and magenta boxes in panel **J**). *N* = 8 animals. **(L)** Response properties of each zone to static deflections. Different letters in blue, red, and magenta in panels **(F,L)** indicate significant differences between stimuli in zones A, B, and E, respectively (Friedman's test followed by Scheffe's multiple comparison for each zone, *P* < 0.05). Statistical analysis in panel **(I)** was performed by Mann-Whitney U-test with respect to each zone. ^***^P < 0.001. ns, not significant. A, anterior; D, dorsal; M, medial. Scale bar in panels **(D,G,J)** = 25 μm.

Electrostatic actuation of the antennal receiver is used to record the compound action potentials in JO neuronal axons of fruit flies in response to movement of the receiver (Albert et al., 2007; Effertz et al., 2012). Compound action potential responses to the receiver's movement elicited by electrostatic force and by sound broadcast from a loudspeaker are indistinguishable with respect to both amplitude and phase (Albert et al., 2007). Based on this study, we developed a method that allows for GCaMP3-based calcium imaging in the brain while actuating the receiver electrostatically to achieve *in vivo* activity imaging of subgroup-D JO neurons (Figure 2A). Using the GAL4/UAS binary expression system, the genetically encoded calcium sensor GCaMP3 (Tian et al., 2009) was expressed in subgroup-D JO neurons. To prevent muscle-based movements of the fly during imaging, its head and body were immobilized by burying the dorsal half of the fly in silicone grease. A living fly with its mouthpart pulled-out to obtain optical access to the AMMC was mounted on an imaging plate (see Materials and Methods for details). The antennal receiver, a feathery arista coupled with the antennal third segment, was kept freely moving. On this preparation, we placed a stimulus electrode close to the arista (< 300 μm) and inserted a charging electrode into the fly's thorax (Figure 2A). When the fly was positively charged, positive charge applied to the stimulus electrode induced deflection of the arista to the posterior side, and negative charge applied to the stimulus electrode deflected the arista to the anterior side (Figure 2B). We next examined whether our electrode limits the force transfer up to 800 Hz by measuring stimulus-induced vibrations of a passive object, the arista of *tilB*^1^ mutant flies (Riabinina et al., 2011), using a Laser Doppler vibrometry. When sinusoidal vibrations were applied to a stimulus electrode, the arista of *tilB*^1^ fly vibrated in a sinusoidal pattern at the same frequency as the applied stimulus with virtually the same displacement amplitudes for the 40, 100, 200, 400, and 800 Hz stimuli (Figure 2C). This result clearly showed that our electrode had a frequency limit more than 800 Hz.

Previous monitoring of the response of JO neurons revealed that subgroups A and B are vibration-sensitive neurons, whereas subgroups C and E are deflection-sensitive neurons (Kamikouchi et al., 2009; Yorozu et al., 2009). To verify whether our electrostatic method could yield the consistent results with the previous reports, we measured responses of the AMMC zones A, B, and E, which are projection targets of subgroups-A, -B, and -E JO neurons, respectively, to vibrations and to static deflections. We used the *F-GAL4* driver (Kim et al., 2003) to express GCaMP3 in essentially all JO neurons.

First, we measured the response of each zone to sinusoidal vibrations. Previous studies showed that vibration-sensitive subgroups have different frequency preferences; subgroup-A JO neurons preferentially respond to high-frequency (peak approximately 400 Hz) sinusoidal vibrations whereas subgroup-B JO neurons preferentially respond to low-frequency (<100 Hz) vibrations (Kamikouchi et al., 2009; Yorozu et al., 2009). Our electrostatic stimulus induced essentially the same response properties; responses of zones A and B peaked at sinusoidal vibrations of high and low frequencies, respectively, as when exposed to sound (Figures 2D–F). On the other hand, zone E did not show strong response to sinusoidal vibrations, which corresponds well with previous reports (Kamikouchi et al., 2009; Yorozu et al., 2009) (Figures 2D–F).

Males of many *Drosophila* species produce a courtship song to attract females. The songs of *D. melanogaster* comprise a short sine component followed by bursts of pulse components, which are called the sine song and pulse song, respectively (Ewing and Bennet-Clark, 1968; Tauber and Eberl, 2003). Subgroups-A and -B JO neurons reportedly respond to the pulse song (Kamikouchi et al., 2009; Yorozu et al., 2009). Consistent with these reports, the AMMC zones A and B showed significant responses to an artificial pulse song carrying a 35-ms IPI. On the other hand, the zone E showed no response to the pulse song (Figures 2G–I).

Finally, we examined responses of zone A, B, and E to static deflections. As reported previously (Yorozu et al., 2009), zone E was selectively activated by posterior deflection of the antennal receiver; no response was observed to anterior deflection (Figures 2J–L). On the other hand, zones A and B showed no response to both of anterior and posterior deflections (Figures 2J–L). Taken together, these results validated our electrostatic method to identify the response properties of JO neurons in the brain.

### Response of subgroup-D JO neurons to vibrations

**Figure 3:**
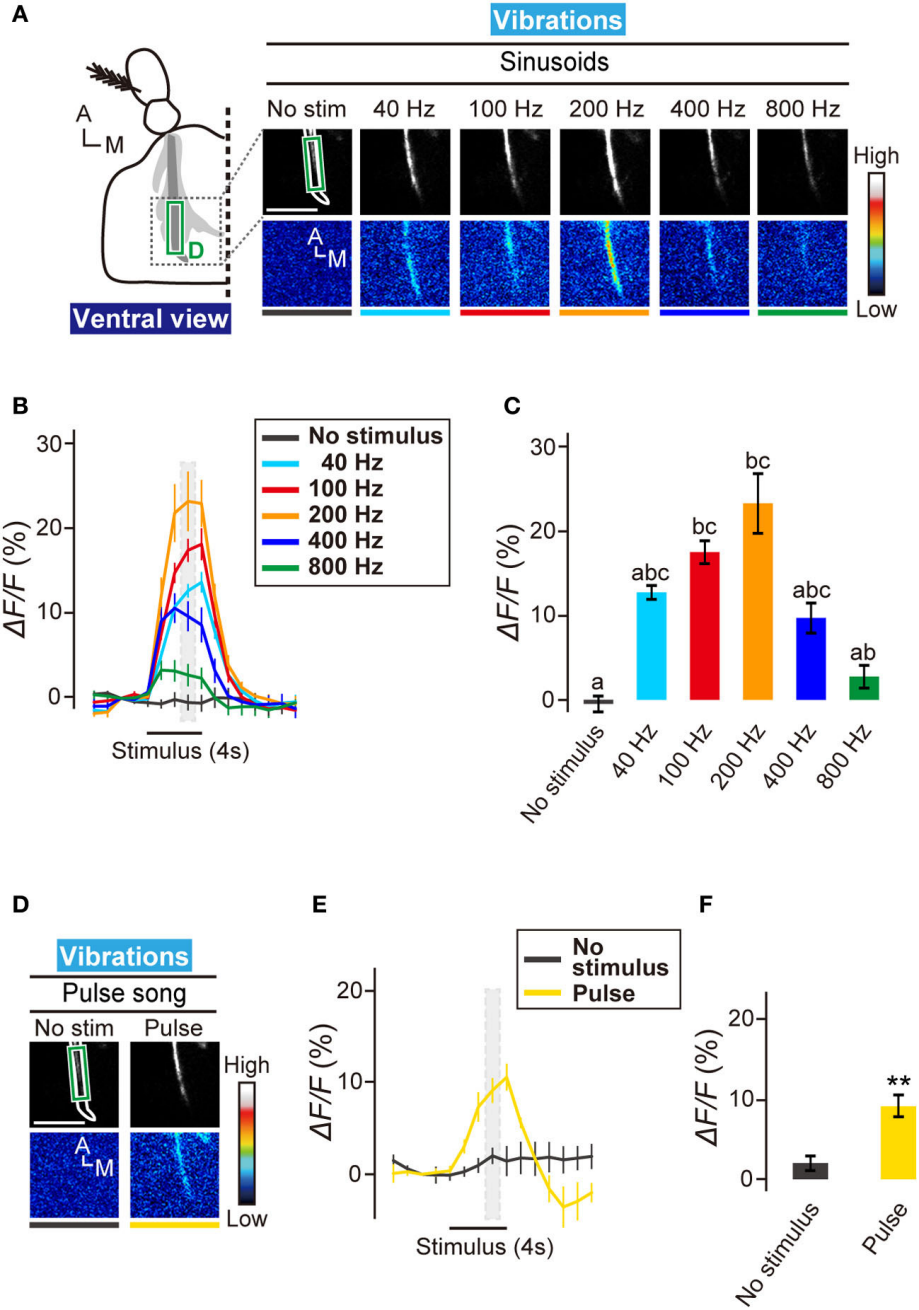
**Neural activity of subgroup-D JO neurons to vibrations. (A)** Calcium response in AMMC zone D to sinusoidal vibrations at 40, 100, 200, 400, and 800 Hz. The region of interests (green box) for calculating changes in fluorescent intensities shown in panels **(B,C)** was set on the main output sites of zone D. Scale bar = 25 μm. **(B)** Time trace of calcium responses to sinusoidal vibrations in zone D (green box in panel **A**). Gray hatched boxes indicate time-windows for calculating Δ*F/F* in no-stimulus and during-stimulus periods shown in panel **(C)**. *N* = 7 animals. **(C)** Response properties of zone D to sinusoidal vibrations. Different letters indicate significant differences between groups (Friedman's test followed by Scheffe's multiple comparison, *P* < 0.05). **(D)** Calcium response in AMMC zone D to a courtship pulse song. Scale bar = 25 μm. **(E)** Time trace of calcium responses to a courtship pulse song in zone D (green box in panel **D**). *N* = 5 animals. **(F)** Response properties of zone D to a courtship pulse song. Statistical analysis was performed by Mann-Whitney U-test, ^**^P < 0.01. A, anterior; M, medial.

To visualize the activity of subgroup-D JO neurons in the brain, we selectively expressed GCaMP3 in subgroup-D JO neurons using the JO29 *GAL4* driver (Kamikouchi et al., 2006). Application of sinusoidal vibrations carrying different frequencies revealed that responses of zone D to vibrations at 100 and 200 Hz showed a significant difference from that of no-stimulus condition (Figures 3A–C). Together, subgroup-D JO neurons comprise vibration-sensitive neurons that prefer middle-range frequencies around 100–200 Hz.

The mean frequencies of sine and pulse songs are 127 and 176 Hz, respectively (Riabinina et al., 2011), which correspond well to the preference of subgroup-D JO neurons. To test whether subgroup-D JO neurons respond to the pulse song, we actuated the antennal receiver with the artificial pulse song carrying a 35-ms IPI. Zone D showed a significant calcium response to this artificial pulse song (Figures 3D–F), suggesting that the vibration-sensitive subgroup-D JO neurons could be involved in perception of the fruit fly courtship song.

### Response of subgroup-D JO neurons to deflections

**Figure 4:**
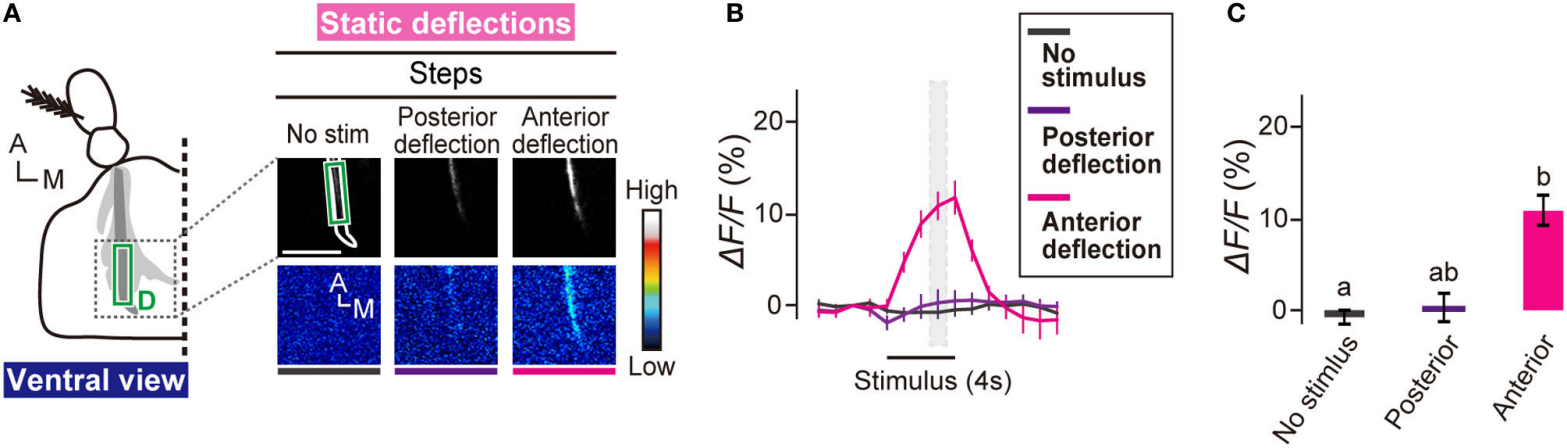
**Subgroup-D JO neurons respond to anterior deflection. (A)** Calcium response in AMMC zone D to static deflections of the antennal receiver. Scale bar = 25 μm. **(B)** Time trace of calcium responses in zone D (green box in panel **A**). Gray hatched boxes indicate time-windows for calculating Δ*F/F* in control and during-stimulus periods shown in panel **(C)**. *N* = 8 animals. **(C)** Calcium response of zone D to static deflections. Statistical analysis was performed by Friedman's test followed by Scheffe's multiple comparison, *P* < 0.05. A, anterior; M, medial.

Subgroup-C and -E JO neurons respond to static deflections of the antennal receiver (Kamikouchi et al., 2009). Interestingly, zones C and E, the projection targets of C and E JO neurons, show a directional sensitivity (Yorozu et al., 2009); anterior and posterior deflections of the receiver activated ipsilateral zones C and E, respectively. We monitored the response of subgroup-D JO neurons to static deflections. Anterior deflection of the receiver induced a strong calcium response in zone D (Figures 4A–C). Posterior deflection, on the other hand, induced essentially no response in zone D (Figures 4A–C). This finding clearly indicates that subgroup-D JO neurons comprise deflection-sensitive neurons that selectively respond to the anterior deflection of the antennal receiver. Taken together, our findings indicate that subgroup-D JO neurons comprise both vibration- and deflection-sensitive neurons.

### Neural circuits downstream of zone D

**Figure 5:**
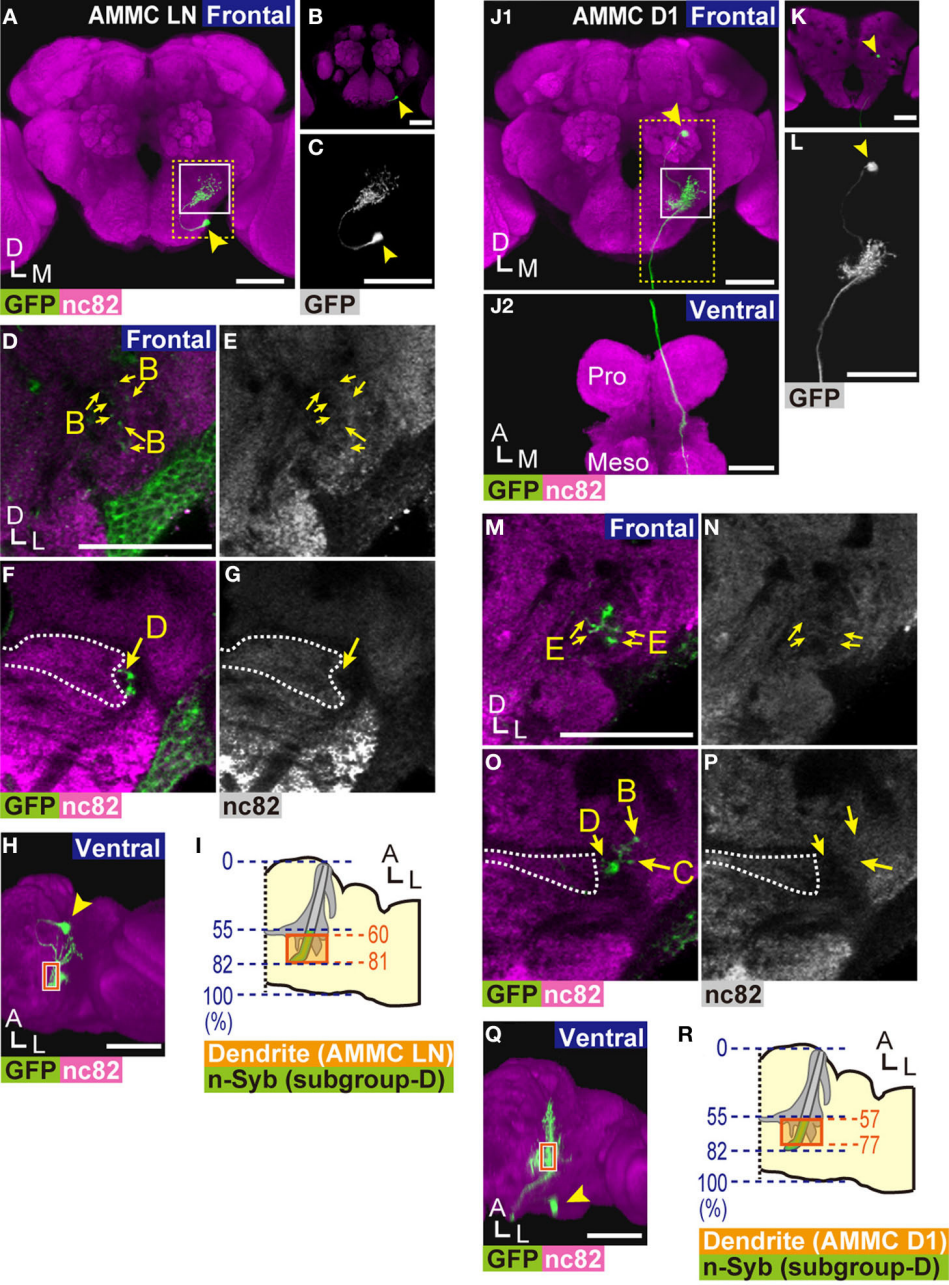
**Projection neurons innervating zone D. (A,B)** 3D-reconstructed frontal images of AMMC LNs in the brain. AMMC LNs cell bodies are located at the anterior side of the brain **(B)**. **(C)** Magnified view of an AMMC LN (yellow dotted box in panel **A**). **(D–G)** Innervation pattern of an AMMC LN. Magnified view of the AMMC (white box in panel **A**). AMMC LNs project to zones B (arrows in panels **D,E**) and D (arrows in panels **F,G**). **(H,I)** Distribution of dendritic regions of AMMC LNs. Ventral view of panel **A (H)**. Blue dotted lines indicate the length ratio along the anterior-posterior axis of the brain **(I)**. Presynaptic sites of subgroup-D JO neurons identified at Figure 1V are shown in green. Orange boxes in panels **(H,I)** show the area where dendrites of AMMC LNs were observed in zone D. **(J,K)** 3D-reconstructed images of AMMC D1 neurons. Arrowheads indicate the position of the cell body. Frontal view of the brain **(J1)** and ventral view of the ventral nerve cord **(J2)** are shown. The axon of an AMMC D1 neuron innervates the prothoracic ganglion (Pro) and mesothoracic ganglion (Meso). Cell bodies of AMMC D1 neurons are located at the posterior side of the brain **(K)**. **(L)** Magnified view of an AMMC D1 neuron (yellow dotted box in panel **J1**). **(M–P)** Innervation pattern of an AMMC D1 neuron in the AMMC. Magnified views of the AMMC (white box in panel **J1**) are shown. AMMC D1 neurons innervate zone E (arrows in panels **M,N**, sections that correspond 40% of the length of the anterior-posterior axis of the brain are shown) and zones B–D (arrows in panels **O,P**). **(Q,R)** Distribution of dendritic regions of AMMC D1 neurons. Ventral view of panel **J1 (Q)**. Blue dotted lines indicate the length ratio along the anterior-posterior axis of the brain **(R)**. Orange boxes show the area where dendrites of AMMC D1 neurons were observed in zone D. Presynaptic sites of subgroup-D JO neurons identified at Figure 1V are shown in green. Arrowheads in panels **(A–C,H,J–L,Q)** indicate the position of the cell body. White dotted lines in panels **(F,G,O,P)** indicate the boundary of zone E. Anti-GFP signals are shown in green in panels **(A,B,D,F,H,J,K,M,O,Q)**, and in white in panels **(C,L)**. nc82 signals are shown in magenta in panels **(A,B,D,F,H,J,K,M,O,Q)**, and in white in panels **(E,G,N,P)**. Distributions of AMMC zones were identified by the staining pattern of nc82 antibody. Scale bar = 50 μm. A, anterior; D, dorsal; M, medial; L, lateral.

To identify the projection neurons that innervate zone D, we screened a collection of 3939 *GAL4* fly strains (NP- and MZ-series; Ito et al., 1995; Hayashi et al., 2002) and selected three strains that label neurons likely innervating AMMC zone D. To precisely map the projection target of these neurons, we used a FLP-out recombination technique (Wong et al., 2002) that allows labeled neurons to be visualized at the single-cell level. The area at the posterior side of the AMMC, where signals of nc82 antibody were sparse (Figures 1E–H,J–M,O–R), was used as a landmark structure for the zone D contour in the AMMC. Two types of neurons extended neuronal fibers into zone D: AMMC local neurons (AMMC LNs, identified by Lai et al., 2012) and AMMC D1 neurons (Figure 5).

#### AMMC local neurons (LNS)

Lai et al. (2012) previously described AMMC LNs as local neurons that distribute only in the unilateral AMMC. Because a detailed projection pattern of AMMC LNs has not yet been identified, we used the NP2228 *GAL4* driver to visualize a single AMMC LN (Figures 5A–C). The AMMC LN cell body, approximately 5 μm in diameter, was located at the anteroventral region of the gnathal ganglia (GNG, previously called the subesophageal ganglion) (arrowhead in Figures 5A–C). From the cell body, the AMMC LN neuronal fiber extended to AMMC zones B and D (arrows in Figures 5D–G), the former of which receives low frequency vibrations detected by JO (Kamikouchi et al., 2009; Figure 2). Input regions of AMMC LNs are likely distributed in the AMMC because the fibers projecting to AMMC zones B and D form dendrite-like neurites (Figures 5D–G). To examine whether the AMMC LN dendrites in zone D are distributed on the output sites of subgroup-D JO neurons, we mapped the location of the dendritic regions of the AMMC LN in zone D along the anterior-posterior axis of the brain (Figures 5H,I). The AMMC LN dendrites were distributed between 60 and 81% of the length of the anterior-posterior axis of the brain (the average of four samples) (Figure 5I), which corresponds well with the distribution of the nSyb::GFP signal on subgroup-D JO neurons (Figure 1V). Output sites of subgroup-B JO neurons reportedly distribute throughout zone B (Kamikouchi et al., 2006). Collectively, our results strongly suggest that the AMMC LNs are downstream of subgroup-B and -D JO neurons, likely to be involved in processing information about antennal movement, which is encoded by subgroup-B and -D JO neurons.

#### AMMC D1 neurons

NP5056 and NP7365 *GAL4* drivers labeled neurons connecting zone D with the ventral nerve cord in the thorax. The cell body of these neurons, which we termed AMMC D1 neurons, was approximately 5 μm in diameter at the posterior side of the brain (arrowhead in Figures 5J–L). Single-cell analysis revealed that AMMC D1 neurons extend long axons ipsilaterally to the posterior side of zone D (Figure 5J1,L), and run through the posterior side of the GNG until finally reaching the dorsolateral region of the ventral nerve cord (Figure 5J2). In the brain, AMMC D1 neurons have intensive dendritic collateral branches innervating zones B, C, D, and E in the ipsilateral AMMC, forming dendrite-like neurites (Figures 5M–P). The AMMC D1 dendrites in zone D were distributed between 57 and 77% of the length of the anterior-posterior axis of the brain (the average of three samples) (Figures 5Q,R), which corresponds well with the anterior side of the region where the nSyb::GFP signal on subgroup-D JO neurons was observed (Figure 1V). These results clearly showed a possible synaptic connection between AMMC D1 neurons and subgroup-D JO neurons.

The output sites of JO neurons reportedly distribute abundantly throughout zones B and C (Kamikouchi et al., 2006). On the contrary, the anteriormost subarea of zone E, subarea EA, was essentially devoid of presynapses of JO neurons, whereas other subareas of zone E (EDM, EVM, EDC, and EDP) carried abundant output sites of JO neurons (Kamikouchi et al., 2006). To estimate the potential synaptic connections between subgroup-E JO neurons and the AMMC D1 neurons, we mapped the relative location of the dendritic regions of the AMMC D1 in zone E along the anterior-posterior axis of the brain. Subarea EA, which lacks abundant presynaptic sites in zone E, reportedly located between 36 and 46% of the length of the anterior-posterior axis of the AMMC (Kamikouchi et al., 2006). Because the posterior end of the AMMC corresponds the posterior end of the AMMC zone D, we estimated the relative position of subarea EA along the anterior-posterior axis of the brain; by multiplying 36 and 46% by 0.82, which is the relative position of the posterior end of the AMMC zone D along the anterior-posterior axis of the brain (Figure 1V), we estimated that subarea EA would distribute between 30 and 38% of the length of the anterior-posterior axis of the brain. The AMMC D1 dendrites in the AMMC zone E, on the other hand, were distributed between 32 and 73% of the length of the anterior-posterior axis of the brain (the average of three samples), which fell into subareas EA, EDM, and EVM (Kamikouchi et al., 2006). These results strongly suggest a spatial overlap between presynapses of subgroup-E JO neurons and the AMMC D1 dendrites. Taken together, the AMMC D1 is likely to be a downstream neuron of subgroups- B, C, D, and E JO neurons. Whereas zone B is a primary center for vibration stimuli, zones C and E receive information about gravity and wind detected by JO (Kamikouchi et al., 2009; Yorozu et al., 2009). AMMC D1 neurons are thus likely involved in the transmission of multiple types of mechanosensory information to the thorax, such as sound, gravity, and wind, detected by one side of the JO.

## Discussion

Electrostatic actuation of the antennal receiver is used to test the integrity of JO in various types of auditory mutants (Albert et al., 2007; Kamikouchi et al., 2009; Effertz et al., 2012). In the present study, we modified this technique to visualize calcium signals in the projection targets of JO neurons in the brain in response to antennal movement (Figure 2). By using this technique, we identified a novel vibration- and deflection-sensitive subgroup of JO neurons in the fruit fly. This subgroup of JO neurons, subgroup-D JO neurons, responded to vibrations and anterior deflection of the arista. On the other hand, other subgroups of JO neurons, subgroups A, B, C, and E, show strong responses to either vibrations or static deflections (Kamikouchi et al., 2009; Yorozu et al., 2009). Zone D in the AMMC is a unique primary center of JO that responds to both types of antennal movements. There are at least two possible explanations for this response property: One is that subgroup-D JO neurons are functionally subdivided into two types of neurons, each of which selectively responds to vibrations or to anterior deflection. The other is that the same set of subgroup-D JO neurons responds to both vibrations and anterior deflection. Many types of sensory neurons in femoral chordotonal organs in stick insects and locusts are sensitive to both position and movement (Field and Matheson, 1998). Considering that JO is a chordotonal organ, it is possible that all or some subgroup-D JO neurons are sensitive to both vibrations and static deflections. Calcium imaging of subgroup-D JO neurons at a single-cell level is necessary to identify if either of these explanations account for this response property.

Zones A and B in the AMMC, the projection targets of subgroup-A and -B JO neurons, respectively, are the primary auditory centers in the fly brain that selectively respond to antennal vibrations imposed by sound stimuli (Kamikouchi et al., 2009; Yorozu et al., 2009); zones A and B receive high and low frequency vibrations, respectively. Here, we found a vibration-sensitive property of zone D; these neurons preferentially respond to middle-range frequency vibrations that peaked around 100–200 Hz. In *D. melanogaster* (Canton S), the mean frequencies of two components of the fly's courtship song, the sine and pulse songs, are 127 and 176 Hz, respectively (Riabinina et al., 2011). Subgroup-D JO neurons could possibly be involved in sensing the courtship song of male flies. Indeed, zone D responded to an artificial pulse song with a 35-ms IPI and a 167-Hz interpulse frequency (Figures 3D–F), which effectively induces courtship behavior in males (Yoon et al., 2013). On the other hand, the flight tone generated by the wing beat of a flying *D. melanogaster* has mean frequency of approximately 145–220 Hz (von Schilcher, 1977; Warmke et al., 1992; Riabinina et al., 2011). This frequency range of wing tone also corresponds well with the response properties of zone D (Figures 3A–C). Thus, subgroup-D JO neurons might also be involved in controlling flight motion based on the flight tone generated by the beating of its own wings. It remains to be examined whether silencing or activating subgroup-D JO neurons affect the courtship and/or flight behaviors or not. Because JO29 *GAL4* strain labels not only JO neurons but also other sensory neurons such as visual and olfactory neurons (data not shown), identification of GAL4 strains that specifically label subgroup-D JO neurons is needed for analyzing behavioral consequences of the activation of subgroup-D JO neurons.

Zones C and E in the AMMC, the projection targets of subgroup-C and -E JO neurons, respectively, are the primary gravity/wind detection centers in the fly brain (Kamikouchi et al., 2009; Yorozu et al., 2009); these neuronal subgroups selectively respond to antennal deflections imposed by gravity and wind. Interestingly, zones C and E are activated by arista deflections in different directions; displacing the arista posteriorly activates zone E, whereas displacing it anteriorly activates zone C (Yorozu et al., 2009). Here, we found that anterior deflection, but not posterior deflection, activated zone D (Figure 4). This finding indicates that zones C and D together constitute a primary center for anterior movement of the arista, whereas zone E is a primary center for posterior movement of the arista.

We identified two types of neurons downstream of zone D, AMMC LNs and AMMC D1 neurons, in the brain (Figure 5). Although whether the innervations of these neurons in zone D have connections with vibration-sensitive and/or deflection-sensitive subgroup-D JO neurons remains to be examined, our study revealed that (1) AMMC LNs, previously identified as local interneurons within the AMMC (Lai et al., 2012), have arborizations in the ipsilateral AMMC zones B and D, (2) the AMMC D1 neurons arborize more broadly and innervate zones B, C, D, and E in the ipsilateral AMMC and send a long projection to the ventral nerve cord, and (3) input regions of both AMMC LNs and AMMC D1 neurons are likely distributed in the AMMC (Figures 5D,E,K,L). These findings suggest that AMMC LNs are likely involved in information processing, possibly by linking two types of antennal movement, whereas AMMC D1 neurons are involved in transmitting information of different mechanosensory modalities detected by the antennal ear to the thorax.

Previous studies had revealed excellent correspondence between stimulus-evoked electrophysiological responses in sensory neurons and calcium responses in their cell bodies and axon termini in olfactory and gustatory receptor neurons of fruit flies (de Bruyne et al., 2001; Suh et al., 2004; Pelz et al., 2006; Kwon et al., 2007; Cameron et al., 2010). Although we do not know the correlation between the calcium signals observed in the axons of subgroup-D JO neurons and their action potential responses, the tonic calcium response of subgroup-D JO neurons to vibrations of middle-range frequencies, a pulse song, and the anterior deflection observed in this study strongly suggests these signals would be transmitted to their downstream neural circuits. A lack of calcium signals to the posterior deflection of the arista, on the other hand, does not necessarily mean that the neurons do not respond to this stimulus with a change in the firing rate of action potentials (Mank et al., 2008). Further studies are thus needed to identify a quantitative correlation between stimuli and complex action potential responses in axons of subgroup-D JO neurons that might be evoked by different types of mechanosensory stimuli.

**Figure 6:**
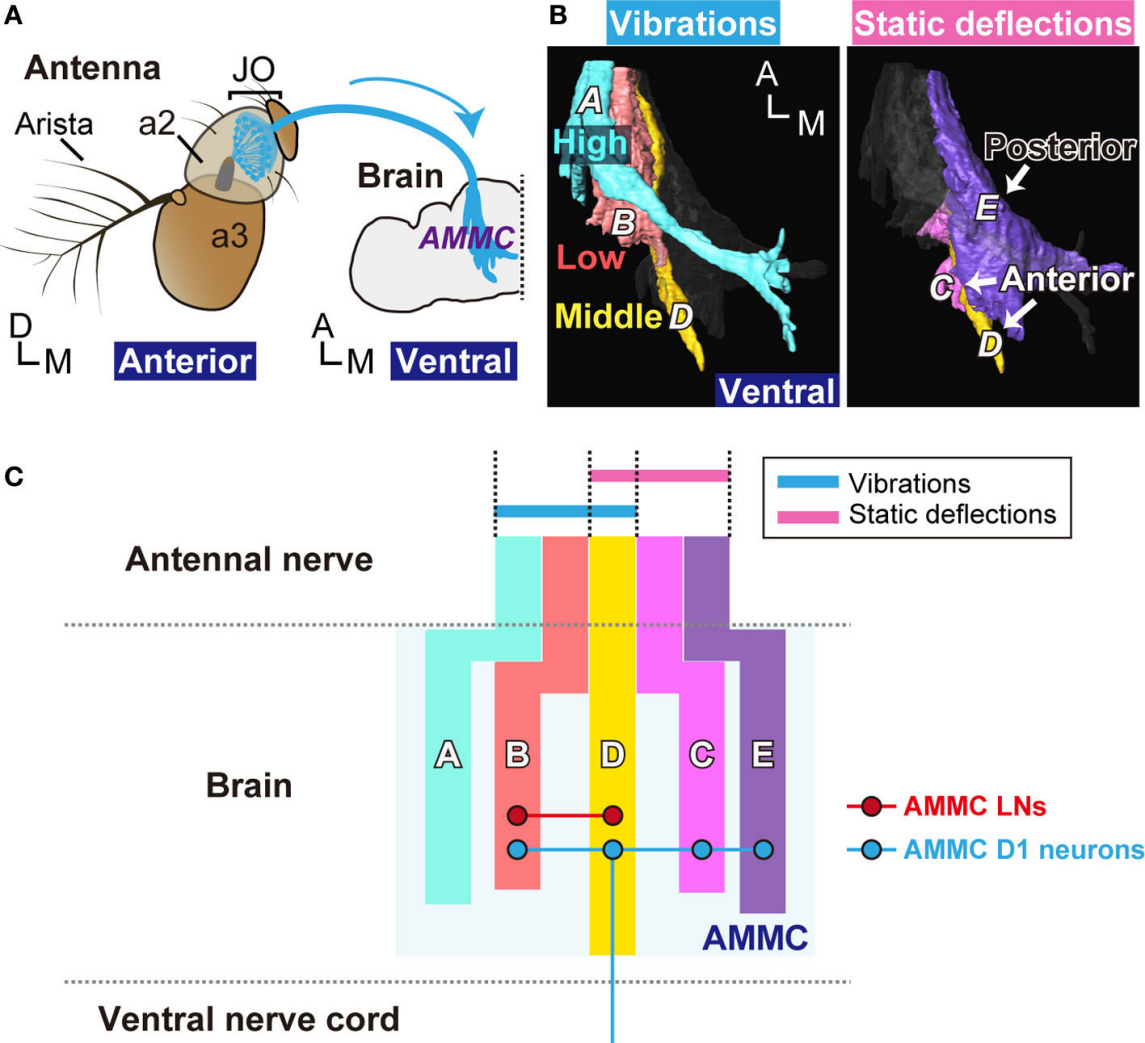
**Neural pathway from Johnston's organ to the brain. (A)** Antennal ear of the fly. Vibrations of the antennal receiver, the arista, and the antennal third segment (a3), are transmitted to JO neurons (blue) in the antennal second segment (a2). JO neurons project their axons to the antennal mechanosensory and motor center (AMMC) in the brain. Modified from Kamikouchi et al. (2006). **(B)** Axonal projections of JO neurons in the brain. Left panel; Zones A (light blue), B (red), and D (yellow), which comprise the primary centers for antennal vibrations (movements), selectively respond to high-, low-, and middle-frequencies, respectively. Right panel; Zones C (pink), D (yellow), and E (purple), which comprise the primary center for antennal deflections (positions), respond to either anterior or posterior deflections. **(C)** Representation of the antennal movement in the fly brain. Color vertical bars show schematic representation of zones in the AMMC (light blue, zone A; red, zone B; yellow, zone D; pink, zone C, and purple, zone E). AMMC LNs (red horizontal line) and AMMC D1 neurons (blue horizontal and vertical lines) project within the AMMC. AMMC D1 neurons also project to the ventral nerve cord. Circles with red and blue lines indicate the putative dendritic targets of AMMC LNs and AMMC D1 neurons, respectively. A, anterior; D, dorsal; M, medial.

In summary, our results clearly showed that subgroup-D JO neurons could encode the position (anterior deflection) and movement (vibrations around 100–200 Hz) of the antennal receiver. The five anatomically-defined zones as projection targets of JO neurons are now defined as three functionally distinct groups: (1) a primary vibration center (zones A and B), (2) a primary deflection center (zones C and E), and (3) a primary vibration and deflection center (zone D) (Figure 6). A comparison of activity patterns among these functional groups could provide a basis for encoding information about complex movements of the antennal receiver, which is activated by mechanical energy imposed on the antenna.

### Conflict of interest statement

The authors declare that the research was conducted in the absence of any commercial or financial relationships that could be construed as a potential conflict of interest.
